# Predictors of Arrhythmic Events Detected by Implantable Loop Recorders in Renal Transplant Candidates

**DOI:** 10.5935/abc.20150106

**Published:** 2015-11

**Authors:** Rodrigo Tavares Silva, Martino Martinelli Filho, Giselle de Lima Peixoto, José Jayme Galvão de Lima, Sérgio Freitas de Siqueira, Roberto Costa, Luís Henrique Wolff Gowdak, Flávio Jota de Paula, Roberto Kalil Filho, José Antônio Franchini Ramires

**Affiliations:** 1 Instituto do Coração do Hospital das Clínicas da Faculdade de Medicina da Universidade de São Paulo, São Paulo, SP - Brazil; 2 Unidade de Transplante Renal - Divisão de Urologia do Hospital das Clínicas da Faculdade de Medicina da Universidade de São Paulo, São Paulo, SP - Brazil

**Keywords:** Arrhythmias, Cardiac, Myocardial Contraction, Kidney Transplantation, Difibrillators, Electric Countershock

## Abstract

**Background:**

The recording of arrhythmic events (AE) in renal transplant candidates (RTCs) undergoing dialysis is limited by conventional electrocardiography. However, continuous cardiac rhythm monitoring seems to be more appropriate due to automatic detection of arrhythmia, but this method has not been used.

**Objective:**

We aimed to investigate the incidence and predictors of AE in RTCs using an implantable loop recorder (ILR).

**Methods:**

A prospective observational study conducted from June 2009 to January 2011 included 100 consecutive ambulatory RTCs who underwent ILR and were followed-up for at least 1 year. Multivariate logistic regression was applied to define predictors of AE.

**Results:**

During a mean follow-up of 424 ± 127 days, AE could be detected in 98% of patients, and 92% had more than one type of arrhythmia, with most considered potentially not serious. Sustained atrial tachycardia and atrial fibrillation occurred in 7% and 13% of patients, respectively, and bradyarrhythmia and non-sustained or sustained ventricular tachycardia (VT) occurred in 25% and 57%, respectively. There were 18 deaths, of which 7 were sudden cardiac events: 3 bradyarrhythmias, 1 ventricular fibrillation, 1 myocardial infarction, and 2 undetermined. The presence of a long QTc (odds ratio [OR] = 7.28; 95% confidence interval [CI], 2.01–26.35; p = 0.002), and the duration of the PR interval (OR = 1.05; 95% CI, 1.02–1.08; p < 0.001) were independently associated with bradyarrhythmias. Left ventricular dilatation (LVD) was independently associated with non-sustained VT (OR = 2.83; 95% CI, 1.01–7.96; p = 0.041).

**Conclusions:**

In medium-term follow-up of RTCs, ILR helped detect a high incidence of AE, most of which did not have clinical relevance. The PR interval and presence of long QTc were predictive of bradyarrhythmias, whereas LVD was predictive of non-sustained VT.

## Introduction

Patients with end-stage renal disease undergoing chronic hemodialysis have a high mortality rate^1^. In 2010, the annual report of the United States Renal Data System (USRDS) showed a mortality rate of 210 deaths per 1000 patient-years, mainly attributable to cardiovascular disease. The single largest cause of death among dialysis patients is sudden cardiac death (SCD), accounting for 27% of all-cause mortality, and it is linked to arrhythmic mechanisms^1-3^.

The particular vulnerability of dialysis patients to SCD has been investigated earlier. Factors including obstructive coronary artery disease, left ventricular hypertrophy, electrolyte shift, and abnormal myocardial ultrastructure and function have been implicated in the increased risk of arrhythmia-mediated death. However, the relative contribution of individual factors to the overall hazard of SCD is still undertermined^1,2,4-7^.

In contrast, it has been previously reported that arrhythmic events (AE) are quite common in dialysis patients. To date, knowledge about the incidence of AE has been limited to detection by conventional resting electrocardiogram (ECG) and electrocardiographic ambulatory monitoring systems^8-12^. However, documentation obtained by these traditional tools at randomly selected intervals may have resulted in huge gaps in temporal knowledge of the incidence of AE. Furthermore, the spontaneous and circadian variabilities of cardiac arrhythmias may have influenced their detection, and thus, its occurrence in dialysis patients may have been underdiagnosed. To date, studies using long-term cardiac monitoring to detect AE in dialysis patients have not been conducted.

The implantable loop recorder (ILR), with automatic detection of arrhythmias, allows for continuous detection, quantification, and documentation of AE during a long-term ECG monitoring period that extends to 36 months. Current clinical use and research with ILR have focused mostly on patients with syncope or ventricular dysfunction after myocardial infarction ^13,14^. The PRETRANSPLANT (Predictors of Arrhythmic Events Detected by ILRs in Hemodialysis Renal Transplant Candidates) study is a prospective observational study conducted to evaluate the incidence and predictors of AE occurrence by using long-term ILR monitoring in renal transplant candidates (RTCs) on hemodialysis.

## Methods

### Study Cohort Selection and Study setting

This study was conducted in a prospective ambulatory cohort of patients with end-stage renal disease (ESRD)referred to the Heart Institute (InCor) of the University of São Paulo Medical School, São Paulo, Brazil, for pretransplantation cardiovascular evaluation between March and December of 2009. A total of 383 consecutive outpatients awaiting a renal transplant were screened during routine clinical assessment. All of the patients who met the inclusion criteria of being on chronic hemodialysis therapy, having a high cardiologic risk profile for renal transplant surgery (defined by age > 50 years, diabetes, or clinically evident cardiovascular disease) and with < 2 years of baseline cardiovascular assessment were eligible for study participation (n = 176)^15^. From this cohort of included patients, 76 patients were further excluded because of refusal to participate (n = 63), planned cardiovascular intervention (n = 6), and previous renal transplantation (n = 3). Three patients died while waiting for ILR implantation and 1 patient was excluded because of inadequate R-wave amplitude measured in a preimplantation ILR test. Finally, 100 patients (mean age, 59 ± 8,8 years; men, 65%; white, 73%) underwent long-term ECG monitoring by ILR and were included in this analysis.

A comprehensive clinical and cardiovascular investigation was carried out for pretransplantation cardiovascular evaluation, including 12-lead resting ECG, signal-averaged electrocardiogram (SAECG), myocardial perfusion scintigraphy, and transthoracic echocardiogram conducted on interdialytic days.

Surface ECG parameters included the heart rate, PR interval, QRS duration, and QT interval. Values of corrected QT interval (QTc) were obtained by measuring the distance of QT interval on 12-lead ECG and then correcting using Bazett’s formula. QT dispersion was calculated by the difference from the longest to the shortest QT interval. A PR interval ≥ 200 ms, a QRS duration ≥120 ms, and a QTc interval > 450 ms (men) or > 470 ms (women) were considered abnormal^16^. Four patients were excluded from QTc analyses due to left bundle branch block, atrial fibrillation (AF), or atrial flutter.

All patients provided written informed consent, and the local institutional ethics committee approved the study protocol (registration number: 1099/08). All phases of the study were free from interference by the ILR manufacturer, including study design, data collection, analysis, and description of the results. This company participated only in donating the devices used during the study.

### ILR implantation and programming

This study used the *Reveal*^®^ XT 9529 Implantable Cardiac Monitor (Medtronic Inc., Minneapolis, MN, USA), a 9 cm^3^, 15 g, subcutaneously implantable device for long-term ECG monitoring that is designed to continuously monitor cardiac rhythm for up to 3 years. The device is usually implanted, under local anesthesia, in the left parasternal region in the area indicated by a specific tool (Vector check).

The ECG data storage can be manually initiated or automatically triggered when AE fulfill the preprogrammed cut-off criteria: asystole (pause lasting >3 s); bradyarrhythmias (4 ventricular events <40 beats/min), ventricular tachycardia (5 ventricular events >120 beats/min), and accelerated ventricular tachycardia/ventricular fibrillation (9 ventricular events >200 beats/min from the past 12 events). AF and atrial tachycardia (AT) were automatically detected by the device using an internal algorithm programmed to detect AT/AF with default parameters. The dedicated AF detection algorithm uses irregularity and incoherence of RR intervals to identify and classify patterns in ventricular conduction. RR intervals were analyzed within each 2-min period, and the difference in duration between consecutive RR intervals (∆R-R) was calculated. Subsequently, the variability of these ∆R-R intervals is calculated, much as a Lorenz plot is constructed. When the RR intervals within the 2-min period show a certain pattern of irregularity, the heart rhythm in this period is classified as AF^17^.

The device memory can automatically hold 28 AE with 30 s of preactivation and 27 s of postactivation ECG storage as well as 3 patient-activated events with 6.5 min of preactivation and 1 min of postactivation ECG storage. The sensitivity of AE detection was adjusted individually according to the number of false events observed at each visit. Patients were followed for at least 1 year after ILR implantation; the first evaluation occurred 2 weeks after hospital discharge and at 8-week intervals thereafter.

### Outcomes

The outcomes were AE detected by ILR during the follow-up period. Each stored AE episode was analyzed and classified by a researcher as bradyarrhythmias and supraventricular or ventricular arrhythmias.

The bradyarrhythmias (heart rate < 40 beats/min) were classified as sinus pause, bradycardia, or atrioventricular (AV) block. Supraventricular arrhythmias were classified as premature atrial beats, non-sustained AT (an abrupt increase in heart rate [> 120 beats/min] lasting < 30 s), or sustained AT/AF (detected by the device according to irregularities in the RR intervals as noted on a Lorenz plot)^17^. Ventricular arrhythmias were categorized as premature ventricular beats, non-sustained ventricular tachycardia (VT), sustained VT (lasting > 30 s), or ventricular fibrillation.

### Statistical analysis

The sample size was estimated as 92 patients based on the reported ventricular arrhythmia prevalence of 40% in a previous study published by Bozbas^18^, with a 10% variation in precision of the absolute estimate and a 95% reliability interval to analyze the proposed objectives and correlations^19^. One patient was excluded due to localized infection 1 month after implantation. Statistical analysis of AE recorded by this ILR was conducted until device removal.

The incidence of AE was described in absolute and relative numbers, and continuous variables were expressed as mean ± standard deviation. Normality was assessed by means of the Kolmogorov-Smirnov test. Comparison of baseline characteristics of patients with and without AE was done with *t* tests for continuous variables and the chi-square test and Fisher’s exact test for categorical variables.

Multivariate logistic regression models using stepwise selection were applied to determine the independent association of baseline characteristics and AE incidence. A 2-sided p value of 0.05 was considered statistically significant and 95% confidence intervals (CIs) are presented for all odds ratios (ORs). All statistical analyses were conducted using a commercially available statistical software package (SPSS version 15.0 for Windows).

## Results

One hundred patients were followed-up during a period of 424 ± 127 days after implantation of ILR, and no data were lost. All devices were inserted during a period of 8 months. Only 1 patient withdrew from the study due to a localized infection after 1 month of implantation, requiring removal of the ILR and antibiotic treatment. The mean duration of hemodialysis treatment was 53.8 ± 30 months. Hypertension was the most common comorbidity (97%), and 84% of patients were on beta-blockers. ECG findings demonstrated sinus rhythm in 99% of the patients, and 1 patient was in AF. The mean heart rate was 73 ± 15 beats/min, and 8% of patients had an intraventricular block. A prolonged QTc interval and an abnormal SAECG were documented in 33% and 3% of the patients, respectively. Echocardiographic characteristics showed mean left ventricular ejection fraction (LVEF) of 59 ± 10%, and 21% of patients had LVEF ≤50%. Abnormal myocardial scintigraphy was noted in 38% of the patients. Baseline clinical and demographic characteristics of patients are presented in Table 1.

**Table 1:** Clinicai, electrocardiographic, and functional characteristics of the study population

**Characteristics**	**n = 100**
Age in years, mean ± SD (median)	59 ± 8.8 (59.1)
Male gender (%)	65
**Ethnicity (%)**	
White	73
Afro-Brazilian/Asian	21 / 6
Systemic arterial hypertension (%)	97
Diabetes mellitus (%)	70
Dyslipidemia (%)	54
Smoking/obesity (%)	9 / 17
Angina/previous myocardial infarction (%)	30 / 34
Heart failure (%)	27
Cerebrovascular disease/peripheral vascular insufficiency (%)	13/55
Duration of hemodialysis in months, mean ± SD (median)	53.8 ± 30 (48.3)
**Medical therapy (%)**	
Angiotensin-converting enzyme inhibitor or angiotensin receptor blockers	50
Beta-blockers/calcium channel antagonists	84 / 31
Acetylsalicylic acid/statins	84 / 62
Amiodarone	1
**Conventional electrocardiogram (n = 100)**	
Heart rate (beats/min)	73 ± 15.2
Sinus rhythm/atrial fibrillation	99% / 1%
PR interval (ms)	173.2 ± 24
QRS (ms)	91.4 ± 17.5
First-degree atrioventricular block	11%
Right bundle branch block/left bundle branch block	4% / 4%
Mean QTc interval (ms)/prolonged QTc (%)	436.4 ± 27.6 / 33%
QT dispersion (ms)	50.7 ± 24.7
Signal-averaged ECG positive (%), n = 100	3%
**Echocardiogram (n = 99)**	
Left atrium (mm), mean ± SD	40.6 ± 6.4
Interventricular septum/posterior wall (mm), mean ± SD	11.7 ± 2 / 11 ± 1.7
Left ventricular diastolic diameter/left ventricular systolic diameter (mm), mean ± SD	50.5 ± 6.1 / 34 ± 6.6
Left ventricular ejection fraction (%), mean ± SD	59.5 ± 10.8
Left ventricular ejection fraction ≤ 50% (%)	21
Cardiac mass index (g/m^2^), mean ± SD	125.2 ± 29.7
Diastolic dysfunction (%)	78
Segmental alterations (%)	26
**Myocardial perfusion scintigraphy (n = 89)**	
Normal/altered/ischemic (%)	62 / 27 / 11

SD: Standard deviation; ECG: Electrocardiogram.

### Incidence and factors associated to AE

**Figure 1:**
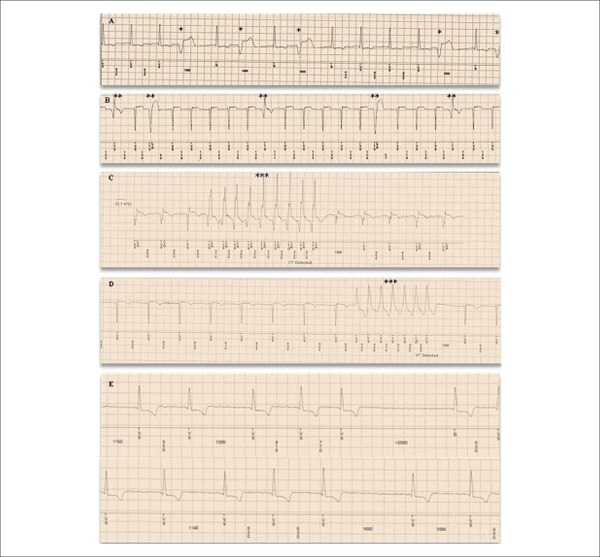
Examples of arrhythmic events recorded by implantable loop recorder during study follow-up. The tracing recorded in (A) shows isolated ventricular premature beats (*); the record in (B) shows polymorphic ventricular premature beats (**); episodes of non-sustained ventricular tachycardia are plotted in (C) and (D) (***), and an episode of atrial fibrillation is recorded in (E).

During the ILR monitoring period, 98% of patients presented AE, with 92% of them presenting AE of more than one type. A total of 5075 AE were detected by ILR, resulting in a rate of 51.79 AE per patient. Supraventricular (94%) and ventricular arrhythmias (79%) were the most frequently diagnosed AE. Table 2 shows a descriptive panel of AE detected by ILR, and some examples of morphology are shown in Figure 1.

**Table 2:** Descriptive panel of arrhythmic events (AE) detected by implantable loop recorder in the study population

**AE **	**Incidence (n = 100)**	**AE (number)**	**AE Rate/pts**
**Follow-up: 424.7 ± 127.1 days**			
Bradyarrhythmias	25%	155	6.20
Asystole	4%	12	3.00
Bradycardia	24%	141	5.87
Advanced atrioventricular block	1**%**	2	2.00
Supraventricular arrhythmias	94%	3.702	39.38
Premature atrial beats	40%	276	6.90
Sinusal tachycardia	69%	1.972	28.58
Non-sustained atrial tachycardia	74%	1.362	18.41
Sustained atrial tachycardia	7%	50	7.14
Atrial fibrillation	13%	42	3.23
Ventricular Arrhythmias	79%	1.218	15.42
Premature ventricular beats	70%	947	13.53
Non-sustained ventricular tachycardia	56%	270	4.82
Sustained ventricular tachycardia/ventricular fibrillation	1**%**	1	1.00

All clinical baseline, electrocardiographic, and functional characteristics were evaluated considering the occurrence of different types of AE, and predictors were identified exclusively for bradyarrhythmias and non-sustained VT. None of these variables was associated with the occurrence of AF or other supraventricular arrhythmias.

According to the AE detected by ILR, we observed that patients with bradyarrhythmias were significantly older (62.7 ± 6.9 years vs. 57.7 ± 9.1 years, p = 0.014), had lower mean HR (69.6 ± 8.7 beats/min vs. 75.5 ± 8.9 beats/min, p = 0.005), and had higher prevalence of coronary artery disease (CAD; 87% vs. 56%, p = 0.026) than those without bradyarrhythmias. Moreover, the PR interval was longer (190.2 ± 32.1 ms vs. 167.5 ± 17.6 ms, p < 0.001); there was a higher prevalence of first-degree AV block (36% vs. 3%, p < 0.001) and prolonged QTc interval (52% vs. 28%, p = 0.028) in patients with bradyarrhythmias. In contrast, there was no significant difference between patients with or without non-sustained VT, except that patients with non-sustained VT were less obese (9% vs. 27%, p = 0.01) and had higher prevalence of LV dilatation (31% vs. 14%, p = 0.04).

**Figure 2:**
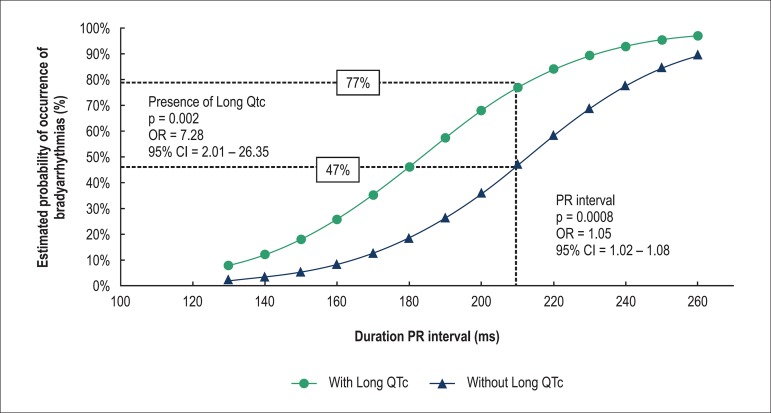
Probability of bradyarrhythmia occurrence detected by implantable loop recorder in relation to PR interval duration stratified according to the type of QTc manifestation (with or without long QTc).

In a multivariate logistic regression model (Table 3), bradyarrhythmias were independently associated with PR interval duration (OR, 1.05 [95% CI, 1.02-1.08], p = 0.0008) and long QTc interval (OR, 7.28 [95% CI, 2.01-26.35], p = 0.002). When a cut-off of 200 ms for PR interval was used (first-degree AV block), the probability of bradyarrhythmias was 77% and 47% for patients with or without long QTc interval, respectively (Figure 2).

**Table 3:** Multivariate analysis demonstrating predictive factors for occurrence of arrhythmic events detected by the implantable loop recorder

	**Bradyarrhythmias**			**Non-sustained ventricular tachycardia**		
	**OR**	**95% CI**	**p value**	**OR**	**95% CI**	** p value**
Prolonged QTc	7.28	2.01-26.35	0.002	-	-	-
Duration of PR interval	1.05	1.02 to 1.08	0.0008	-	-	-
Left ventricular dilatation	-	-	-	2.83	1.01-7.96	0.041

OR: Odds ratio; CI: Confidence interval.

Furthermore, the presence of LV dilatation (OR, 2.83, [95% CI = 1.01-7.96], p = 0.04) was the only independent factor associated with non-sustained VT. Patients with LV dilatation were 2.8 times more likely to have non-sustained VT than those without it.

### Clinical findings

**Figure 3:**
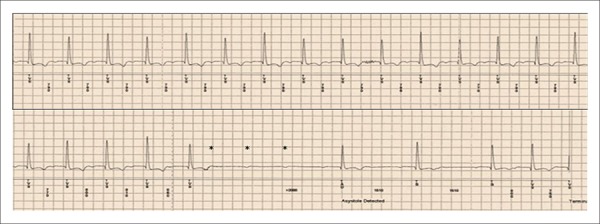
Recording of an advanced atrioventricular block episode by the implantable loop recorder. The marks (*) show three blocked P waves, indicating a pause of 3.1 s.

**Figure 4:**
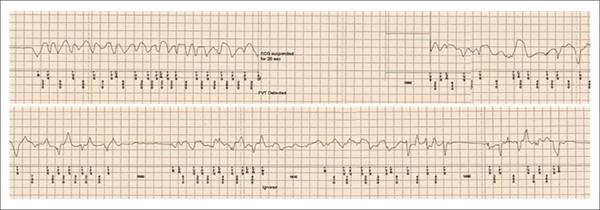
Fatal ventricular tachycardia/fibrillation episode recorded by the implantable loop recorder.

Despite almost all patients (98%) presenting some type of AE, most had no significant clinical implication. All patients with AE reported at least 1 episode of palpitations, and no patient had syncope. Potentially serious bradyarrhythmias and ventricular tachyarrhythmias occurred in 4 and 1 patient, respectively. Three patients died suddenly due to bradyarrhythmia, and a patient with dilated cardiomyopathy developed advanced AV block (Figure 3), requiring pacemaker use and removal of the ILR. A 47-year-old man without structural heart disease died because of a malignant ventricular arrhythmia (Figure 4).

During the study follow-up period, 18 patients died, of whom 7 had SCD, 1 had heart failure (HF), and 10 died of non-cardiac causes. Among the cases of SCD, there were 3 bradyarrhythmias, 1 ventricular fibrillation, 1 acute myocardial infarction, and, in the remaining 2 cases, the ILR could not be analyzed and the causes of death have not been identified. Other nonfatal major cardiovascular events included acute pulmonary edema (9 patients), acute myocardial infarction (2 patients), acute peripheral vascular disease (2 patients), and ischemic stroke (1 patient). In addition, 14 patients underwent renal transplantation.

## Discussion

In the PRETRANSPLANT study, continuous cardiac monitoring provided by ILR in RTCs on hemodialysis revealed a high incidence of arrhythmias. This new technology has recently been incorporated in this clinical setting, providing a prolonged period of cardiac monitoring that allows determination of relevant clinical and electrocardiographic correlations and solves the problem of spontaneous and circadian variabilities in AE. In addition, we identified several clinical factors independently associated with the most prevalent types of AE in the dialysis population. Importantly, supraventricular arrhythmias had no significant clinical implications; in contrast, bradyarrhythmias and ventricular arrhythmias were associated with serious and fatal clinical events.

Our findings showed the occurrence of at least some type of AE in 98% of patients. It is difficult to compare this study to others because none of the previous studies used an ILR for diagnosing cardiac arrhythmias. Normally, the ILR has been used to evaluate patients with recurrent syncope and a less severe cardiac risk profile^13^. Moreover, previous studies using 24-h Holter monitoring for the detection of AE in the dialysis population reported a highly variable occurrence rate. Bozbas et al^18^ showed that 85.2% of 94 hemodialysis patients experienced an AE. Similar results were observed by Sforziniet al^20^, who detected an AE in 76% of 127 hemodialysis patients. In contrast, D’Elia et al^11^ reported AE in 26.2% of 122 patients, and 1 patient had an advanced AV block, and we observed the same rate in our study. This variability in the incidence of AE is most likely dependent on the specific characteristics of the population and on the different diagnostic criteria used for each study.

Many factors contributing to the increased incidence of AE in dialysis patients have been identified. Several studies have shown the influence of structural cardiac disease^9,21,22^, HF^11,23,24^, electrolyte abnormalities^8,10^, postdialysis hypokalemia^10^, low potassium and calcium concentrations in the dialysate solution^8^, silent ischemia related to dialysis^24^, the use of digoxin^8,10^, the presence of left ventricular hypertrophy^8,21^, hyperparathyroidism, and uncontrolled hypertension^22,25,26^; however, there are no studies that used continuous electrocardiographic monitoring methods. The present study showed that alterations in the conventional ECG and echocardiogram were independently associated with the AE detected by the ILR.

Previous studies reported that electrocardiographic alterations, such as prolonged PR and QT intervals, have been considered predictive factors for cardiovascular events and mortality in the end-stage renal disease and general populations^27,28^. In dialysis patients, all of these findings were more evident and have been associated with AE^12,27,29^. Abe et al^12^ showed that first-degree AV block is more prevalent in patients with chronic kidney disease undergoing hemodialysis (5.4%) than in those who were not receiving dialysis (1.4%) and in healthy controls. Hage et al^27^ identified prolonged PR interval on conventional ECG in 15% of patients, and this was not associated with worse survival. In our study, the prevalence of first-degree AV block was 11% and it was 12 times more prevalent in patients with bradyarrhythmias. For each 10-ms increase in the PR interval duration, the likelihood of a bradyarrhythmia increased by 5% during the follow-up.

Other studies have shown that the QT interval is significantly longer in dialysis patients than in healthy subjects. Furthermore, it has been demonstrated that the QT interval increases during dialysis and is associated with AE^27-32^. In our study, the mean QTc interval (436.4 ± 27.6 ms) was similar to the results reported by Suzuki et al^29^ (432.6 ± 24.9 ms) and slightly lower than those observed in the study by Hage et al^27^ (447 ± 35 ms). It is noteworthy that all of these measurements are within the normal range (≤450 ms in men and ≤470 ms in women)^16^. Despite this, there was a high prevalence of long QTc in our study (33%), and this was similar to the results obtained by Hage et al.**(39%)^27^. This is in agreement with the data in the literature, wherein the increase in the QT interval and the presence of a long QT have been associated with AE occurrence. Therefore, a careful evaluation using conventional ECG should be done routinely in this population, whereby these patients with higher risk of AE can be stratified.

Another interesting finding in our study was that LV dilatation occurred in 23% of patients and almost tripled the occurrence of non-sustained VT. Parfrey et al^33^ found that in a study of 432 patients, the prevalence of LV dilation (28%) was slightly higher than that in our study, and it was associated with CAD, hypertension, anemia, and hypoalbuminemia. Previous studies have shown that ventricular dilatation is a marker of poor prognosis in the dialysis population and is associated with LV volume overload^30,33-35^. The eccentric hypertrophy induced by this mechanism causes cardiomyocyte stretching, apoptosis, and cell death, which results in myocardial fibrosis. The combination of these factors with neurohormonal hyperactivity and HF may precipitate the occurrence of AE^36-39^. Thus, an echocardiographic evaluation to diagnose structural changes may help identify patients at high risk of arrhythmias in the dialysis population.

Predictors of AE occurrence is a subject that is not well explored in the literature. Bozbas et al^18^ showed a predictive relationship between hypertension, CAD, and QT dispersion with complex ventricular arrhythmias. Moreover, they observed a causal relationship between the duration of dialysis therapy and premature atrial contraction. More specifically, Nishi et al^40^ identified the age and presence of P-terminal force >0.04 mm/s as predictors of new-onset AF. Distinctly, our study could not establish any independent predictors of AF occurrences, but has identified predictors of bradyarrhythmia, which affected 3% of our patients, and non-sustained VT.

In summary, a conjunction of various risk factors may provide a very favorable scenario for the high occurrence of AE in the dialysis population. Moreover, taking into account the random characteristic of the occurrence of AE, and that its documentation requires absolute synchronism, it is important to emphasize that prolonged monitoring with an ILR may play a role in prospective investigations aiming to reduce cardiovascular events in this high-risk population.

### Study Limitations

Some aspects of this analysis merit further consideration. First, the study population comprised consecutive hemodialysis patients derived from an academic institute rendering routine tertiary healthcare with interdisciplinary follow-up. Therefore, caution must be exercised when extrapolating these findings to other RTCs.

Second, the ILR may have some limitations related to detection properties; for example, the electrocardiographic analysis is taken from an isolated, single, subcutaneous ECG lead, and atrial depolarization is difficult to identify. The algorithm used by the device to identify AF expends 2 min in observation of RR-interval irregularity distribution in a Lorenz plot, which imposes a limit of inability to autoidentify AF episodes shorter than this duration. Another limitation of the ILR is a memory capacity limited to 30 AE recordings. When the memory was saturated, a new AE recording replaced the oldest, a feature known as loop memory recording. In patients with a high frequency of cardiac arrhythmias, this feature limits the AE recordings, and thus, results in underestimation of their real incidence. In the present study, it issue was minimized by reducing the intervals between the visits to 8 weeks. However, 40% of our patients had saturated ILR memory, which could have prevented analysis of certain types of AE.

## Conclusions

The PRETRANSPLANT study is the first to report medium-term monitoring of AE recorded by an ILR in a high-risk population of RTCs. Most AE detected were not clinically relevant, but serious bradyarrhythmias and/or ventricular tachyarrhythmias occurred. Defining clinical characteristics that can stratify patients at higher risk of AE can help prevent complications or identify patients who require stricter monitoring. In our study, the factors independently associated with AE were the PR interval and the presence of long QTc for bradyarrhythmias and LV dilatation for non-sustained VT.
